# The Burden of Cow’s Milk Protein Allergy in the Pediatric Age: A Systematic Review of Costs and Challenges

**DOI:** 10.3390/healthcare13080888

**Published:** 2025-04-12

**Authors:** Rita Nocerino, Greta Aquilone, Stefania Stea, Teresa Rea, Silvio Simeone, Laura Carucci, Serena Coppola, Roberto Berni Canani

**Affiliations:** 1 Department of Translational Medical Science, University of Naples “Federico II”, Via S. Pansini 5, 80131 Naples, Italy; gr.aquilone@studenti.unina.it (G.A.); laura.carucci@unina.it (L.C.); serena.coppola3@unina.it (S.C.); berni@unina.it (R.B.C.); 2 ImmunoNutritionLab at CEINGE Advanced Biotechnologies, University of Naples “Federico II”, Via S. Pansini 5, 80131 Naples, Italy; 3 Department of Biomedicine and Prevention, University of Rome “Tor Vergata”, 00133 Rome, Italy; 4 Master’s Degree Course in Nursing and Midwifery Sciences, Faculty of Medicine, University Nostra Signora del Buon Consiglio, 1000 Tirana, Albania; s.stea10594@stud.unizkm.al; 5 Department of Public Health, University of Naples “Federico II”, 80131 Naples, Italy; teresa.rea@unina.it; 6 Department of Clinical and Experimental Medicine, University of Catanzaro Magna Graecia, 88100 Catanzaro, Italy; silvio.simeone@unicz.it; 7 European Laboratory for the Investigation of Food-Induced Diseases, University of Naples “Federico II”, 80131 Naples, Italy; 8 Task Force for Microbiome Studies, University of Naples “Federico II”, 80131 Naples, Italy

**Keywords:** cow’s milk allergy, economic burden, healthcare costs, hypoallergenic formulas, multidisciplinary management, insurance reimbursement

## Abstract

**Background.** Cow’s Milk Protein Allergy (CMPA) is a prevalent pediatric food allergy affecting 2–5% of infants globally. CMPA presents significant clinical and economic challenges, requiring specialized diagnostic procedures, dietary management with hypoallergenic formulas, and multidisciplinary care. The financial burden on families and healthcare systems includes direct costs (e.g., diagnostic tests, medical consultations, and formula expenses), indirect costs (e.g., caregiver absenteeism and productivity loss), and intangible costs (e.g., psychological distress and impaired quality of life). Economic disparities further exacerbate these challenges, particularly in low-resource settings where access to specialized care is limited. **Methods.** A systematic review was conducted following PRISMA guidelines using PubMed, CINAHL, Scopus, and Cochrane databases to identify studies on the economic and psychosocial burden of CMPA. Studies published between 2010 and 2024 were analyzed. From an initial search of 11,565 articles, 802 duplicates were removed, leaving 10,763 articles for title and abstract screening. Twenty full-text articles meeting the inclusion criteria were included in the final analysis. Thematic categories focused on direct, indirect, and intangible costs, with findings synthesized to highlight global disparities and policy gaps. **Results.** CMPA management imposes substantial economic burdens, with hypoallergenic formulas alone accounting for up to 15% of household income in some regions. Indirect costs, including lost workdays and additional childcare expenses, further strain families. Intangible costs, such as heightened caregiver anxiety and reduced social participation, are significant yet frequently overlooked. Healthcare system disparities, particularly regarding insurance coverage for diagnostic tests and therapeutic formulas, contribute to financial inequities. The use of extensively hydrolyzed casein formulas with probiotics has demonstrated cost-effectiveness in promoting immune tolerance while reducing healthcare utilization. **Conclusions.** Addressing the economic impact of CMPA would benefit from standardized cost assessment frameworks and equitable access to cost-effective therapeutic options. Further analysis of reimbursement policies across diverse healthcare systems may provide insights into optimizing support for essential treatments. Integrating clinical and economic strategies can alleviate the burden on affected families and optimize healthcare resource allocation. Future research should prioritize longitudinal analyses and cross-regional comparisons to guide sustainable and equitable management strategies.

## 1. Introduction

Cow’s Milk Protein Allergy (CMPA) is one of the most prevalent food allergies in the pediatric population, affecting an estimated 2–5% of infants globally [1,2]. CMPA is an immune-mediated hypersensitivity to cow’s milk proteins, primarily casein and whey, that could manifest through IgE-mediated, non-IgE-mediated, or mixed mechanisms [2,3]. IgE-mediated CMPA typically results in rapid-onset symptoms, such as urticaria, angioedema, vomiting, or anaphylaxis, whereas non-IgE-mediated CMPA is characterized by delayed onset of gastrointestinal and/or cutaneous symptoms [4,5]. These distinct immune and clinical features pathways complicate diagnosis, particularly as non-IgE-mediated symptoms often overlap with other gastrointestinal disorders [6]. The clinical implications of CMPA are profound. In fact, it has been demonstrated that CMPA could be the first step to the progression to other allergic conditions such as eczema, asthma, and rhinoconjunctivitis, the so called “allergic march”, and also to other diseases including functional gastrointestinal disorders (FGIDs), inflammatory bowel diseases (IBD), celiac disease, eosinophilic esophagitis (EoE) [2,7,8,9,10,11]. In addition, evidence has suggested a potential association between CMPA and neuropsychiatric disorders, although further research is needed to clarify this relationship [12]. This underscores the importance of early and effective management to mitigate long-term complications and prevent increased costs associated with managing additional comorbidities [2,7,8,9,10,11,12].

The management of CMPA typically requires the elimination of cow’s milk proteins from the diet of the lactating mother in breastfed infants or the use of hypoallergenic formulas, such as extensively hydrolyzed formulas (eHFs) or amino acid-based formulas (AAFs), in non-breastfed infants [2]. Emerging evidence supports the inclusion of the probiotic *L. rhamnosus* GG (LGG) to accelerate clinical recovery and to promote immune tolerance to cow’s milk proteins [7,8,13,14,15,16,17,18,19,20,21,22,23]. For some children, gradual reintroduction of baked milk through the “milk ladder” approach can enhance dietary variety and improve quality of life [24]. However, these interventions require consistent monitoring of growth and nutritional status to prevent faltering growth and micronutrient deficiencies [2].

Beyond its clinical dimensions, CMPA imposes substantial psychosocial and economic burdens on families. Parents often face significant out-of-pocket expenses for specialized formulas and dietary adaptations, which can account for up to 15% of household income in some settings [25]. Indirect costs, such as lost workdays and the need for additional childcare, further strain family finances, while intangible costs—such as psychological stress, anxiety, and reduced quality of life—are pervasive yet underreported [26]. These challenges are exacerbated in low- and middle-income countries, where access to diagnostic resources and hypoallergenic formulas is limited [27].

Despite growing recognition of these issues [4,26], existing research on CMPA’s economic burden remains fragmented. Most studies are region-specific and fail to account for the diversity of healthcare systems, reimbursement policies, and cultural contexts. Intangible costs, such as the emotional and social impacts of CMPA, are especially underexplored [28]. Addressing these gaps requires a holistic approach that integrates clinical, economic, and societal perspectives. This review aims to bridge these gaps by synthesizing evidence on the multifaceted burden of CMPA. By exploring the interplay between clinical management and economic challenges, the review seeks to illuminate the global implications of CMPA and identify actionable strategies to reduce costs, enhance accessibility, and support affected families more effectively.

## 2. Methodology

### 2.1. Objectives and Review Design

This systematic review was registered in the Open Science Framework and was conducted in accordance with PRISMA guidelines. The review aims to provide a comprehensive assessment of the economic burden associated with CMPA, encompassing direct, indirect, and intangible costs. Given the limited availability of real-world cost analyses across all healthcare systems, our approach is based on a systematic synthesis of the most relevant and methodologically sound studies published between 2010 and 2024. This analysis is based solely on previously published studies, as no original data were collected specifically for this review.

The key objectives are as follows:

1. Identify and quantify financial challenges: to examine the direct and indirect financial burdens of managing CMPA, including medical, dietary, and caregiving costs.
2. Explore broader implications for healthcare and society: to assess the impact of CMPA on healthcare systems, public health policies, and societal welfare, including productivity losses and increased healthcare demands.
3. Examine strategies for cost-effective management: to provide recommendations for improving the cost-effectiveness of CMPA management strategies across diverse healthcare settings, with particular emphasis on interventions that can reduce both clinical and economic burdens [29].

### 2.2. Search Strategy

The literature search was conducted across four major databases: PubMed, CINAHL, Scopus, and Cochrane. Specific keywords were selected to capture a broad range of relevant studies, including “families”, “children”, “cost”, “costs”, “cow’s milk protein allergy”, and “cow’s milk allergy.” Two primary search strings were formulated:

1. ((families AND children) AND (cost OR costs) AND (cow’s milk allergy OR cow’s milk protein allergy))
2. ((families OR family) AND (members OR relatives) AND (children OR adolescents OR youth OR child OR teenager) AND (cost OR costs OR expense) AND (cow’s milk AND protein AND allergy)).

The search included studies published between 2010 and 2024.

This timeframe was selected to ensure that our review reflects the most up-to-date and methodologically robust data on the economic burden of CMPA. Over the past decade, there have been significant advancements in healthcare policies, reimbursement strategies, and the development of new diagnostic and therapeutic approaches for CMPA, all of which have influenced its economic impact. Additionally, economic evaluation methodologies have improved, allowing for more precise assessments of direct, indirect, and intangible costs associated with CMPA [1,2,4,5,7,8,13,15,16,18,19,20,21,25,30]. Given that older studies may not accurately represent current healthcare frameworks and cost structures, we prioritized studies within this timeframe to provide the most relevant insights. Nevertheless, we acknowledge that historical trends can offer valuable context, and we have ensured that our review incorporates key foundational knowledge where applicable.

Articles were imported into the Rayyan software for streamlined organization and analysis.

### 2.3. Selection Criteria

Inclusion Criteria:

- Study type: peer-reviewed and full-length articles, including epidemiological studies, cost analyses, and observational studies.
- Language: articles published in English.
- Publication Date: studies published between 2010 and 2024.

Exclusion criteria:

- Non-peer-reviewed articles, including opinion pieces, editorials, and letters to the editor.
- Conference abstracts and proceedings without full-text availability.
- Studies not explicitly addressing the economic costs or burden of CMPA.
- Non-English language publications where reliable translation was not available.

Unpublished studies, dissertations, or industry-sponsored reports with unclear methodologies.

- Gray literature, including government or institutional reports, unless they provide robust economic data validated by independent sources.

While gray literature can offer valuable insights, particularly on healthcare policies and cost estimates, its inclusion was limited to reports that met high methodological standards and were produced by reputable health organizations or regulatory agencies. The primary reason for excluding most of the gray literature was to maintain consistency in study design and to ensure that all included studies underwent a peer-review process.

### 2.4. Selection Process

The search strategy identified a total of 11,565 articles (6891 from PubMed, 4655 from CINAHL, 18 from Scopus, and 1 from Cochrane), which were imported into Rayyan for screening. After removing 802 duplicates, 10,763 articles remained for title and abstract screening.

**Figure 1:**
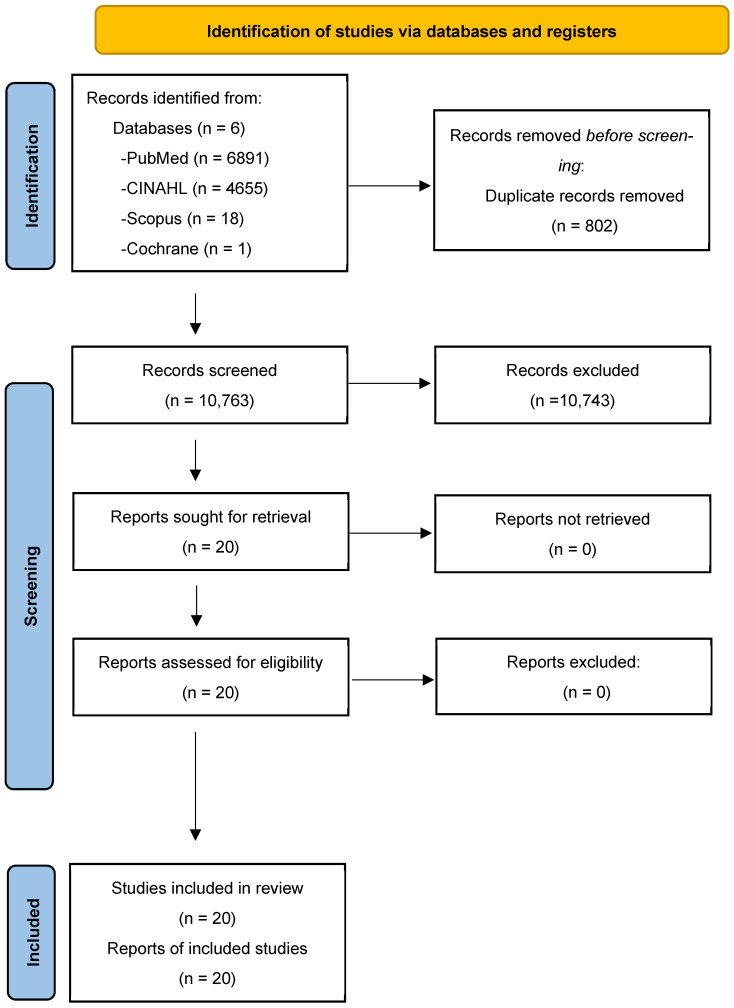
PRISMA diagram.

A total of 20 articles were included in the final analysis. Title and abstract screening were conducted independently by two authors (R.N and St.St.) to ensure objectivity, and disagreements were resolved through discussion. Full-text evaluations further refined the selection, guided by the PRISMA framework to ensure transparency and reproducibility (Figure 1).

### 2.5. Data Extraction and Analysis

Data from the selected studies were extracted and organized into categories, including direct medical expenses, indirect caregiver burden, and intangible psychosocial costs. Analysis involved systematic coding to identify key themes and relationships between variables such as economic disparities and policy gaps. To facilitate comparison across different studies, all cost data have been standardized in US dollars (USD). Where costs were originally reported in other currencies, conversion was performed using historical exchange rates corresponding to the publication year of each study. However, these figures are not adjusted for purchasing power parity (PPP), and cross-country comparisons should consider variations in income levels and healthcare access.

Key interconnections between cost factors, including healthcare access, reimbursement policies, and societal impacts, were explored to provide a holistic understanding of the economic burden of CMPA. Findings are presented in alignment with this study’s objectives to inform evidence-based interventions and policy recommendations.

In Table 1 are reported the characteristics of the eligible studies.

## 3. Results

### 3.1. Direct Costs

The diagnosis and management of CMPA impose significant direct costs on families. Accurate diagnosis often involves consultations with specialists, diagnostic tests such as skin prick tests, serum-specific IgE testing, and oral food challenges. The double-blind placebo-controlled food challenge (DBPCFC), though considered the gold standard for confirming the diagnosis of CMPA in the vast majority of cases, is resource-intensive and impractical in many settings due to the associated costs and time requirements [27]. Prolonged diagnostic timelines, necessitating multiple consultations, further exacerbate financial strain, particularly in complex cases where symptoms overlap with other conditions [6,28].

Once diagnosed, CMPA management relies heavily on hypoallergenic formulas, including eHFs and AAFs. These specialized formulas are crucial for preventing malnutrition and managing allergic symptoms effectively, but are significantly more expensive than standard formulas, posing a substantial financial burden for families [36,37].

In addition, dietary costs extend beyond hypoallergenic formulas, encompassing allergy-friendly food products and fortified options to ensure adequate nutritional intake. These expenses are particularly burdensome for low-income families, often consuming over 15% of monthly household income [5,25].

Families also incur expenses for ancillary medical needs, including medications such as antihistamines, corticosteroids, and epinephrine autoinjectors, which are essential for managing acute allergic reactions and reducing the risk of severe complications [36]. Furthermore, hospital outpatient visits account for the largest expenses [31]. These findings highlight the economic strain on families, particularly given that direct medical costs are significantly higher compared to other allergic conditions like atopic dermatitis or asthma [31].

Education and nutritional counseling further contribute to the financial burden. These services are vital for equipping parents with strategies to avoid allergens, prepare specialized formulas, and ensure adequate nutritional intake for their children. However, such services are often not covered by insurance, adding to out-of-pocket expenses [4]. Psychological support and therapy, while crucial for addressing the stress and lifestyle changes associated with CMPA, represent additional costs that can improve quality of life but also amplify the overall economic impact [37].

### 3.2. Indirect Costs

The management of CMPA imposes substantial indirect costs on both individual caregivers and society at large. Caregivers, often parents, frequently face absenteeism from work due to medical appointments, dietary counseling, and care during acute allergic reactions, which diminishes productivity and, in severe cases, can lead to job instability or loss [31].

These responsibilities have long-term repercussions, including reduced lifetime earnings and limited career advancement opportunities [28,37]. The constant vigilance required to manage CMPA contributes to emotional exhaustion, with caregivers frequently reporting heightened stress and burnout, which further impacts household productivity and overall quality of life [4,6].

At the societal level, reduced workforce participation among caregivers results in macroeconomic losses, particularly in regions with high CMPA prevalence [24]. These losses strain public health and social support systems, amplifying the broader economic burden. Additionally, CMPA significantly impacts healthcare infrastructure. The frequent need for consultations, follow-ups, travel costs, and emergency care escalates resource demands, often diverting attention from other critical healthcare priorities [31]. This challenge is exacerbated by the high costs of specialized treatments and diagnostic procedures, which strain healthcare budgets, particularly in countries with universal healthcare systems [5,26].

### 3.3. Intangible Costs

CMPA places a substantial intangible burden on families, profoundly affecting their emotional well-being, social interactions, and overall quality of life. For parents, the constant vigilance required to prevent accidental exposure to allergens leads to heightened stress, anxiety, and emotional strain. The fear of severe allergic reactions, such as anaphylaxis, exacerbates this burden, impacting caregivers’ mental health and household productivity [4,37].

The emotional toll extends to siblings, who may feel overlooked due to the disproportionate attention required by the affected child. This dynamic can lead to feelings of resentment or neglect, disrupting family harmony and increasing tension [35]. Furthermore, managing CMPA imposes strict dietary restrictions, necessitating careful meal planning and limiting participation in food-centric social events. These adjustments often result in social isolation, affecting not just the child with CMPA but the entire family, further amplifying feelings of exclusion and stress [5,28].

Family dynamics are also significantly impacted. The unequal distribution of caregiving responsibilities frequently creates tension between partners, particularly when one parent assumes the majority of these duties. This imbalance can lead to burnout and strain within relationships [26]. Coordinating care among schools, healthcare providers, and other stakeholders adds another layer of complexity to daily routines, increasing emotional exhaustion.

Additionally, siblings often struggle with feelings of unfair treatment due to the additional attention given to the child with CMPA. These dynamics can foster behavioral challenges and disrupt family cohesion, necessitating psychosocial support to maintain balance within the household [40]. Comprehensive care strategies that address both the psychological and practical dimensions of managing CMPA are essential for alleviating these intangible costs and improving the overall quality of life for affected families [20].

### 3.4. Cost Variations by Region

The financial burden of managing CMPA varies significantly across regions due to differences in healthcare systems, access to hypoallergenic formulas, and financial aid availability [40]. In Australia, the annual healthcare cost of CMA management was estimated to be heavily influenced by the reliance on specialized clinical nutrition preparations, which account for over 60% of total costs [33].

In countries with universal healthcare systems, such as the UK and the Netherlands, families benefit from comprehensive insurance schemes that cover many costs associated with CMPA management. For instance, in the Netherlands, clinical nutrition preparations account for up to 91% of the total cost of CMPA management, with insurers reimbursing a significant portion [40].

In contrast, in countries like the United States, with predominantly private healthcare systems, families face higher overall costs due to inconsistent insurance coverage for hypoallergenic formulas. These gaps result in substantial out-of-pocket expenses, highlighting the need for standardized reimbursement policies [34]. Similarly, in low- and middle-income countries, such as Indonesia and Thailand, access to essential treatments like EHF and AAF is often limited. Families in these regions bear a disproportionate share of costs, further compounded by minimal public support [5,29].

While absolute cost estimates provide insight into the economic burden of CMPA, direct comparisons across countries with different population sizes and healthcare systems are challenging. Future studies should consider standardizing healthcare costs based on GDP or per capita expenditure to improve comparability.

Table 2 summarizes the estimated economic burden of CMPA across different healthcare systems, highlighting both the direct financial impact on families and the total costs borne by national healthcare providers.

### 3.5. Insurance and Reimbursement Policies

Insurance and reimbursement policies are pivotal in shaping the economic burden on families managing CMPA. In Europe, comprehensive insurance coverage, often in some countries, includes hypoallergenic formulas and diagnostic procedures, easing financial pressures on families. For example, in the UK, EHCF + LGG has been demonstrated to be cost-effective, reducing long-term costs for both the National Health Service (NHS) and families [35].

However, in countries where insurance policies exclude hypoallergenic formulas from coverage, such as the United States, families face substantial out-of-pocket costs. In Brazil, while studies show that the use of AAF in diagnostic elimination diets can save costs for the public healthcare system, limited reimbursement options increase the financial strain on families [32].

### 3.6. Economic Disparities

CMPA disproportionately affects low-income families, amplifying existing economic inequalities. The high costs associated with hypoallergenic formulas, dietary adjustments, and frequent medical consultations can consume a significant portion of household income. For example, in Turkey, only 21% of low-income families could adhere to physicians’ recommendations for hypoallergenic formula use due to financial constraints [28].

In low-resource settings, such as South Africa, the lack of government subsidies for hypoallergenic formulas exacerbates disparities. The annual cost of managing CMPA in private-sector patients is estimated to exceed R40,000 (approximately USD 5482 2007/08 exchange rates) per child, making effective treatment unattainable for many middle- and low-income households [38].

### 3.7. Role of Multidisciplinary Teams in Reducing Costs

The effective management of CMPA requires a coordinated multidisciplinary approach, involving pediatricians, allergists, nurses, and dietitians. Pediatricians and allergists play a crucial role in early detection and accurate diagnosis, using advanced tools to minimize unnecessary or repeated testing. These accurate diagnoses not only ensure tailored treatment plans that address the specific needs of the child but also help avoid misdiagnosis and associated long-term complications, ultimately reducing healthcare costs [37].

Nurses and dietitians complement the efforts of medical specialists by providing continuous education, dietary planning, and nutritional counseling. These interventions are vital for managing symptoms, preventing complications such as growth delays or nutrient deficiencies, and reducing reliance on emergency care. Furthermore, the use of cost-effective therapeutic options like EHCF + LGG has demonstrated significant clinical and economic benefits. Multidisciplinary teams are essential in ensuring—when possible—these therapies are effectively integrated into patient care, optimizing resource utilization and improving outcomes while mitigating financial burdens [5,22].

Family education and support are cornerstones of cost-effective CMPA management. Nurses often serve as primary educators, equipping families with practical knowledge about allergen avoidance, symptom recognition, and emergency responses. This education empowers families to manage CMPA effectively at home, reducing the frequency of hospital visits and associated healthcare costs. Dietitians and nurses also provide essential support to address the psychosocial challenges faced by families, such as stress and anxiety, further enhancing adherence to dietary regimens and improving overall quality of life [4,38].

In addition to direct patient care, multidisciplinary teams play a pivotal role in advocating for systemic policy changes to alleviate the financial burden on families. Potential strategies could include expanding insurance coverage for hypoallergenic formulas and diagnostic procedures, exploring the standardization of reimbursement policies across regions, and enhancing public health initiatives to increase CMPA awareness, while considering the differences in healthcare funding models. Cost-effectiveness studies, such as those conducted in the UK, highlight the potential for substantial healthcare savings through comprehensive reimbursement policies for therapeutic formulas [24].

In low- and middle-income countries, advocacy efforts should focus on subsidizing essential treatments and improving access to specialized care to address economic disparities. Expanding subsidy programs for low-income families and enhancing healthcare provider training in CMPA-specific care can further reduce diagnostic delays and optimize management outcomes [5]. By integrating these strategies into healthcare policies, multidisciplinary teams can significantly mitigate the economic and psychosocial burdens of CMPA, ensuring equitable care for affected families.

## 4. Discussion and Future Directions

This review highlights the multifaceted economic burden of CMPA, addressing direct, indirect, and intangible costs. Direct costs, including medical consultations, diagnostic tests, and hypoallergenic formulas, impose significant financial strain on families, particularly in regions with limited healthcare support. Hypoallergenic formulas, especially EHCF + LGG, have demonstrated cost-effectiveness by accelerating immune tolerance and reducing healthcare utilization, thereby alleviating out-of-pocket expenses for families [5,35]. Indirect costs, such as parental absenteeism and reduced workforce productivity, disproportionately affect low-income families and exacerbate economic disparities. Caregiving responsibilities often hinder career advancement and result in long-term financial instability [35]. Intangible costs, including psychological stress, social isolation, and disrupted family dynamics, further compound the economic challenges, emphasizing the need for holistic management approaches [28]. To address these economic and policy gaps more effectively, robust evidence from countries such as the UK, Italy, and France has demonstrated that incorporating EHCF + LGG into public healthcare reimbursement schemes significantly reduces direct medical costs, healthcare resource utilization, and caregiver absenteeism [22,29,35,36,40]. Moreover, evidence from Australia and the Netherlands indicates that comprehensive insurance coverage for hypoallergenic formulas substantially mitigates family financial burdens, suggesting a clear direction for policy intervention in countries lacking standardized reimbursement frameworks [33,40].

A critical limitation in current research is the lack of standardized methodologies for evaluating CMPA-related costs across diverse healthcare systems. Region-specific approaches hinder comparability and generalizability [28]. Developing a globally applicable framework for assessing direct, indirect, and intangible costs is essential to enable meaningful cross-regional comparisons [40]. The cost-effectiveness models discussed in this review were developed within specific healthcare systems, each with distinct reimbursement policies, cost structures, and patient management approaches. While these models provide valuable insights into the economic burden of CMPA and the potential benefits of different management strategies, their applicability to other healthcare settings may be limited. Future research should focus on adapting these models to local healthcare frameworks to enhance their relevance and generalizability. Longitudinal studies are urgently needed to capture the evolving economic and psychosocial burdens of CMPA. Tracking healthcare resource utilization, family productivity losses, and psychosocial toll over time is crucial to understanding cost trajectories, particularly in cases where children develop tolerance or face comorbidities associated with the allergic march [9]. Future studies should also incorporate patient-reported outcomes to better quantify intangible costs, such as emotional distress and reduced quality of life. Another potential limitation of our review is the restriction of included studies to those published from 2010 onwards. This decision was made to prioritize recent and high-quality data reflecting the current healthcare and economic landscape. While older studies may provide historical context, significant changes in CMPA management, healthcare policies, and cost structures over the past decade could render previous economic evaluations less applicable to contemporary settings. Nevertheless, our review ensures a comprehensive synthesis of the most recent evidence, offering a detailed assessment of the direct, indirect, and intangible costs associated with CMPA in different healthcare systems. Lastly, a potential limitation of our review is the availability of economic data across different regions and healthcare systems. While we aimed to capture a broad range of cost-related studies, disparities in healthcare funding models, reimbursement policies, and access to hypoallergenic formulas may have influenced the generalizability of our findings. Additionally, while consulting healthcare professionals from different regions could have provided further insights, our review is based exclusively on published data to ensure methodological consistency. Future studies incorporating real-world cost data from multiple healthcare settings would help refine and expand upon our findings.

Given the substantial economic burden associated with CMPA, implementing cost-effective strategies is crucial to improving accessibility and reducing financial strain on families and healthcare systems. Countries like the UK and the Netherlands have successfully integrated reimbursement programs for hypoallergenic formulas, significantly reducing out-of-pocket expenses. However, in countries like the United States, where insurance policies exclude these formulas, families bear a substantial financial burden [2]. Expanding similar reimbursement policies in nations with inconsistent coverage could significantly improve affordability and access to essential CMPA treatments. The use of EHCF + LGG has been particularly cost-effective compared to AAF, as it reduces healthcare utilization and accelerates immune tolerance [35]. Encouraging the early adoption of these formulas, especially in regions with high CMPA prevalence, could lead to considerable cost savings over time.

The implementation of multidisciplinary care models further contributes to the reduction in healthcare costs by improving early diagnosis and minimizing hospital admissions. Pediatricians, allergists, nurses, and dietitians play a crucial role in ensuring accurate diagnosis and personalized treatment plans, which can prevent misdiagnosis and unnecessary medical interventions [4]. In low-resource settings, direct subsidies for CMPA management could be a viable strategy to address disparities in healthcare access. In South Africa, for example, the lack of government support for hypoallergenic formulas has resulted in management costs exceeding USD 5482 per child annually in private healthcare settings, exacerbating economic inequities [38]. Expanding public health initiatives to include direct subsidies for hypoallergenic formulas and related medical expenses could significantly alleviate financial burdens on families.

The importance of early diagnosis and preventive strategies cannot be overstated, as delayed diagnosis often leads to prolonged symptoms and increased medical expenditures. Public health initiatives that emphasize early screening and standardized diagnostic guidelines could improve timely intervention, preventing unnecessary medical visits and hospitalizations [9]. Additionally, the integration of digital health solutions, such as telemedicine and dietary management programs, could enhance access to specialized care, particularly in remote or underserved regions. Virtual consultations and digital platforms could reduce travel costs and caregiver absenteeism, indirectly alleviating the economic burden associated with CMPA [29,39].

Addressing these challenges requires targeted policy interventions and strategic healthcare reforms. The adoption of cost-effective management strategies could alleviate financial strain while optimizing clinical outcomes. Evidence suggests that expanding access to EHCF + LGG may reduce healthcare utilization and accelerate immune tolerance, although regional differences in healthcare systems and reimbursement models should be considered [5]. Standardizing reimbursement policies across regions is critical to ensuring equitable access to these essential treatments, particularly in low- and middle-income countries where disparities are most pronounced [38].

Multidisciplinary care teams play a pivotal role in managing CMPA effectively and cost-efficiently. Integrating pediatricians, allergists, dietitians, and nurses into care pathways ensures comprehensive management that reduces complications and improves adherence to treatment plans. Educating families on allergen avoidance, symptom recognition, and emergency responses minimizes indirect costs such as lost productivity and caregiver absenteeism [37]. This approach also optimizes resource allocation while enhancing patient outcomes.

Public health initiatives should focus on early diagnosis and awareness campaigns to mitigate treatment delays. Early intervention reduces long-term healthcare costs by preventing the progression of CMPA to other allergic conditions, such as atopic eczema and asthma. Subsidies for hypoallergenic formulas and diagnostic resources can address financial barriers for low-income families, fostering more equitable healthcare access [24].

To advance research, future efforts should focus on developing and validating standardized methodologies for cost assessments across different regions. It is essential to conduct longitudinal studies to capture the evolving economic and psychosocial impacts of CMPA. Additionally, incorporating patient-reported outcomes will allow for a more accurate quantification of intangible costs, such as emotional distress and reduced quality of life. Finally, evaluating the cost-effectiveness of emerging therapies and their implementation in diverse healthcare settings will provide valuable insights for optimizing patient care and resource allocation. The establishment of standardized reimbursement policies for hypoallergenic formulas and diagnostic procedures should be prioritized to alleviate economic disparities, especially in low- and middle-income countries. For example, targeted subsidy programs successfully implemented in European nations have significantly reduced financial inequalities [29,33]. Furthermore, integrating multidisciplinary teams comprising pediatricians, allergists, dietitians, and specialized nurses into the healthcare delivery model has proven cost-effective by improving early diagnosis accuracy, enhancing treatment adherence, and reducing unnecessary medical expenses and caregiver productivity losses [22,35,36]. These integrated care models should be actively promoted as central elements of national and regional policy frameworks.

By addressing these gaps and implementing targeted interventions, healthcare systems can alleviate the multifaceted burden of CMPA on families, fostering equitable access to care and improving patient outcomes globally.

## 5. Conclusions

CMPA represents a substantial clinical and economic burden for the affected families and healthcare systems worldwide. This review highlights the significant direct, indirect, and intangible costs associated with CMPA management, emphasizing the disparities in healthcare access and reimbursement policies across different regions. The high financial strain, particularly in low-resource settings, underscores the urgent need for cost-effective interventions, standardized insurance coverage, and equitable access to hypoallergenic formulas and diagnostic tools [26,28].

To mitigate these impacts, a collaborative effort among policymakers, researchers, and healthcare providers is essential. The development and subsidization of cost-effective interventions, such as hypoallergenic formulas and diagnostic resources, alongside the standardization of insurance coverage and reimbursement policies, could mitigate the economic burden of CMPA. However, these strategies should be tailored to the specific contexts of different healthcare systems [5,35]. Researchers should focus on filling critical gaps in the literature, particularly on the long-term economic and psychosocial burdens of CMPA, and develop robust, globally applicable methodologies for cost assessments [24,25]. Healthcare providers play a pivotal role in delivering multidisciplinary care, integrating dietary management, family education, and psychological support to improve outcomes while reducing costs [35].

Future healthcare policies should therefore prioritize evidence-based reimbursement strategies and multidisciplinary care models, drawing on international experiences to ensure equitable, sustainable, and effective CMPA management.

Finally, addressing the global burden of CMPA requires a holistic and inclusive approach that integrates clinical care with economic and policy innovations. By fostering collaboration and ensuring equitable access to resources, we can alleviate the profound challenges faced by families and enhance the overall quality of care for children with CMPA.

## Figures and Tables

**Table 1:** Characteristics of eligible studies.

Author	Title	Year	Aim	Research Questions/ Hypothesis	Study Design	Methodology	Sample Description	Results	Conclusion	Limitations and Biases
Alanne S et al. [31]	Costs of allergic diseases from birth to two years in Finland	2012	To determine the costs of diagnosing and treating allergic diseases in early childhood.	What are the economic implications of diagnosing and treating allergic diseases during infancy?	Cost-analysis study	Data obtained from an ongoing mother-infant nutrition study, focusing on 60 infants with allergic diseases and 56 healthy infants.	116 infants: 60 with allergic diseases (atopic dermatitis, food allergy, asthma) and 56 without.	CMA emerged as the most expensive allergic disease, significantly impacting families and society.	CMA is the costliest among allergic diseases in infancy, requiring targeted resource allocation.	Conducted in Finland with a publicly funded healthcare system, limiting generalizability. Small sample size and geographically restricted data may not be representative of broader populations. Retrospective cost estimations may introduce recall bias.
Fakih-Botero I. et al. [25]	Out-of-pocket expenses and parent-reported quality of life in children with cow’s milk protein allergy in Bogotá, Colombia	2024	To assess the economic burden and quality of life in children with cow’s milk protein allergy.	Does the economic burden correlate with the quality of life of children with cow’s milk protein allergy?	Cross-sectional study	Parent-reported surveys using the Food Allergy Quality of Life Questionnaire in two tertiary care centers.	122 families of children aged 0–5 with cow’s milk protein allergy.	Median quality of life score was 3.21, and out-of-pocket costs for treatment varied significantly.	The quality of life scores were not directly associated with out-of-pocket expenses, but additional food allergies and older age reduced quality of life.	Relied on self-reported data for out-of-pocket expenses and quality of life, introducing potential recall bias. The sample was limited to Bogotá, Colombia, affecting generalizability. Socioeconomic confounders were not fully addressed.
Cawood A.L. et al. [4]	The health economic impact of cow’s milk allergy in childhood: A retrospective cohort study	2022	To quantify the economic burden of cow’s milk allergy and its management.	How does cow’s milk allergy impact healthcare costs and utilization compared to those without CMA?	Retrospective matched cohort study	Analysis of healthcare data, including prescriptions and healthcare contacts from the UK Health Improvement Network database.	6.998 children: 3.499 with CMA and 3499 without, matched for age and sex.	Children with CMA had significantly higher healthcare costs and required more healthcare contacts than those without CMA.	CMA represents a significant healthcare burden, with implications for resource allocation and management strategies.	Retrospective database study, possibly introducing misclassification errors. Funded by a formula manufacturer (Nutricia Ltd.), which may present a conflict of interest. Findings may not be applicable outside the UK healthcare system.
de Morais M.B. et al. [32]	Amino acid formula as a new strategy for diagnosing cow’s milk allergy in infants: Is it cost-effective?	2016	To evaluate the cost-effectiveness of using an amino acid formula for diagnosing CMA.	Is the use of amino acid formula a cost-effective strategy for diagnosing CMA?	Pharmacoeconomic study	Decision model using TreeAge Pro, informed by expert opinions and literature review.	Infants under 24 months with suspected CMA, analyzed within the Brazilian public healthcare system.	The new strategy was found to be cost-effective, with lower costs and more symptom-free days compared to traditional methods.	The amino acid formula approach offers a dominant, cost-effective strategy for diagnosing CMA.	Decision model based on expert opinion and literature review rather than direct clinical data, potentially leading to bias. Focused on the Brazilian Public Healthcare System, limiting its applicability to other healthcare settings. Assumptions regarding cost-effectiveness may not fully reflect real-world variability.
Fong A.T. et al. [26]	The Economic Burden of Food Allergy: What We Know and What We Need to Learn	2022	To review the current understanding of the economic burden of food allergy and identify knowledge gaps.	What are the primary drivers of the economic burden of food allergy, and where are the knowledge gaps?	Review article	Analysis of studies from various countries, synthesizing economic data, and highlighting gaps.	Studies from the US, UK, France, and others cover food allergies broadly.	Food allergy imposes significant direct and indirect costs, with variations by region and type of allergy.	The economic burden is substantial and multifaceted, requiring further research into prevention and cost-effective management.	Focused heavily on data from the United States, making international comparisons difficult. Differences in healthcare structures and costs limit generalizability. Some economic assessments were based on theoretical models rather than empirical data.
Guest J. F. et al. [33]	Modeling the resource implications and budget impact of managing cow milk allergy in Australia	2009	To estimate the resource and budget impact of managing cow milk allergy in Australia.	What are the resource implications and budget impact of CMA management in Australia?	Decision-modeling study	Decision model constructed with clinical outcomes and resource utilization estimates.	6.150 newly diagnosed CMA infants managed within the Australian healthcare system.	The six-month cost of managing CMA was estimated at AUD 6.5 million, with formulas as the primary cost driver.	CMA management imposes a substantial economic burden; potential efficiencies exist with alternative management strategies.	Modeled healthcare costs based on clinician estimates rather than real-world patient data, which could introduce bias. Conducted in Australia, limiting applicability to other healthcare systems. Cost assumptions may not fully reflect price fluctuations over time.
Guest J. F. et al. [22]	Relative cost-effectiveness of an extensively hydrolyzed casein formula containing Lactobacillus rhamnosus GG in managing infants with cow’s milk allergy in Italy	2015	To estimate the cost-effectiveness of using an extensively hydrolyzed casein formula with Lactobacillus rhamnosus GG.	Is the extensively hydrolyzed casein formula with probiotics cost-effective for CMA management?	Decision-modeling study	Decision model based on observational data and healthcare resource use in Italy.	Infants with IgE-mediated and non-IgE-mediated CMA in the Italian healthcare system.	The formula was cost-effective, improving tolerance development and reducing overall costs compared to alternatives.	The addition of probiotics to hydrolyzed formulas offers cost-effective management for CMA in Italy.	Funded by a commercial entity (Nutricia), raising concerns about potential bias. Cost-effectiveness assumptions may not be directly transferable to other healthcare systems. The study relies on decision modeling, which may not fully capture clinical complexities.
Karakurt T. et al. [28]	Experiences and attitudes of parents of children with cow’s milk and other food allergies	2022	To evaluate the experiences and attitudes of parents managing children with food allergies.	What challenges do parents face in managing children with food allergies, particularly cow’s milk allergy?	Survey-based study	18-item questionnaire completed by 558 parents, focusing on allergy history, diagnosis, and treatment.	558 parents of children with food allergies, primarily cow’s milk allergy.	Delayed diagnosis and difficulties in formula use were reported; 21.1% of patients used hypoallergenic formulas as prescribed.	Improving diagnosis timelines and adherence to prescribed treatments is crucial for better outcomes.	Survey-based study relying on self-reported data from parents, which may introduce recall and reporting bias. The study was conducted in Turkey, so findings may not be generalizable to countries with different healthcare infrastructures. The sample was drawn from an allergy association, potentially introducing selection bias.
Guest J. F. et al. [34]	Cost-effectiveness of using an extensively hydrolysed casein formula containing Lactobacillus rhamnosus GG in managing infants with cow’s milk allergy in the US	2018	To estimate the cost-effectiveness of using an extensively hydrolysed casein formula with Lactobacillus rhamnosus GG in the US.	Is the extensively hydrolysed casein formula with probiotics cost-effective for CMA management in the US?	Decision-modeling study	Decision model using observational data and healthcare costs in the US.	Infants diagnosed with cow’s milk allergy managed within the US healthcare system.	The formula was cost-effective, reducing costs for third-party insurers and parents compared to alternatives.	Adding probiotics to hydrolysed formulas is a cost-effective strategy for managing CMA in the US.	Based on economic modeling rather than a direct clinical trial, which may introduce uncertainty in real-world application. Conducted in the US, limiting its generalizability to other healthcare systems. Assumptions regarding formula adherence and cost structures may not fully reflect actual practice.
GuestJ. F. and Singh H. [35]	Cost-effectiveness of using an extensively hydrolyzed casein formula supplemented with Lactobacillus rhamnosus GG in managing IgE-mediated cow’s milk protein allergy in the UK	2019	To evaluate the cost-effectiveness of a hydrolyzed casein formula with probiotics in the UK.	Does the addition of Lactobacillus rhamnosus GG improve the cost-effectiveness of dietary management for CMA?	Decision-modeling study	Model based on UK healthcare costs and clinical outcomes over a 5-year period.	Infants with IgE-mediated CMA in the UK healthcare system.	The formula significantly reduced healthcare costs and improved outcomes compared to standard formulas.	The use of probiotic-supplemented formulas is cost-effective and beneficial in reducing symptoms and costs.	The study relies on a Markov model using data from an observational study rather than a randomized controlled trial. Economic estimates are based on UK healthcare costs, making comparisons to other settings difficult. Funded by an organization with potential conflicts of interest.
Martins R. et al. [36]	Cost-effectiveness analysis of hypoallergenic milk formulas for the management of cow’s milk protein allergy in the United Kingdom	2021	To compare the cost-effectiveness of hypoallergenic formulas in reducing allergic manifestations and promoting immune tolerance in infants with CMPA.	Hypoallergenic formulas, particularly EHCF + LGG, are more cost-effective in managing CMPA compared to alternatives.	Trial-based decision analytic cohort model	Simulated allergic manifestations in infants with CMPA over 3 years using UK cost resources.	Infants with IgE-mediated symptoms of CMPA in the UK.	EHCF + LGG was cost-effective, reducing healthcare resource use and improving immune tolerance over three years.	EHCF + LGG is the most cost-effective formula for CMPA management in the UK.	Decision-analytic model based on a prospective cohort study, but lacks direct randomization, potentially introducing selection bias. Focused solely on the UK National Health Service (NHS), limiting generalizability to other healthcare systems. Some cost estimates are derived from previous literature rather than direct patient data.
Ngamphaiboon J. et al. [6]	Direct medical costs associated with atopic diseases among young children in Thailand	2012	To estimate the direct medical costs of atopic diseases among children aged 0–5 years in Thailand.	Atopic diseases impose a substantial economic burden on healthcare systems in Thailand.	Cost-of-illness model	Used prevalence-based approach with bottom-up cost analysis for atopic diseases.	Children aged 0–5 years with atopic diseases in Thailand.	Total direct costs estimated at THB 27.8 billion (USD 899 million); CMA had the highest cost per treated patient.	Atopic diseases in young children are a significant financial burden in Thailand.	Conducted in Thailand, where healthcare costs and disease burden may differ from Western countries, limiting global applicability. Cost estimates rely on expert opinion rather than direct hospital data. Lacks consideration of indirect costs associated with atopic diseases.
Ovcinnikova O. et al. [20]	Cost-effectiveness of using extensively hydrolyzed casein formula plus probiotics compared to other formulas for cow’s milk allergy in the US	2015	To assess cost-effectiveness of EHCF + LGG compared to other formulas for CMA management in the US.	EHCF + LGG improves outcomes and reduces costs in CMA management.	Cohort study with economic analysis	Used case records of infants and analyzed costs over 12 months.	Infants under six months with CMA in the US.	EHCF + LGG led to better outcomes and reduced costs compared to AAF and EHCF.	EHCF + LGG is more cost-effective than alternatives for CMA management.	Retrospective analysis of insurance claims data may introduce coding errors or misclassification bias. The study focuses on the US healthcare system, limiting transferability of cost-effectiveness conclusions. Potential conflict of interest due to industry funding.
Paquete T.A. et al. [29]	Managing cow’s milk protein allergy in Indonesia: a cost-effectiveness analysis of hypoallergenic milk formulas from the private payers’ perspective	2022	To assess the cost-effectiveness of various hypoallergenic milk formulas in managing CMPA in Indonesia.	EHCF + LGG is the most cost-effective hypoallergenic formula.	Trial-based decision analytic cohort model	Simulated symptoms and cost outcomes using data from Indonesian clinicians and national databases.	Children with IgE-mediated CMPA in Indonesia.	EHCF + LGG was associated with better outcomes, reduced resource use, and cost savings compared to other formulas.	EHCF + LGG is the most cost-effective formula for CMPA management in Indonesia.	The study is based on a cost-effectiveness model rather than real-world clinical data. The analysis is limited to the perspective of private payers, excluding public healthcare implications. Findings are specific to Indonesia and may not be transferable to other healthcare systems.
Protudjer L.P.J. et al. [37]	Household Costs Associated with Objectively Diagnosed Allergy to Staple Foods in Children and Adolescents	2015	To estimate the direct, indirect, and intangible costs of food allergy in households with children and adolescents.	Food allergies significantly increase household costs compared to controls.	Cross-sectional cost analysis	Used questionnaires to collect parent-reported data on costs and health impacts.	Children and adolescents with food allergies and matched controls.	Households incurred significantly higher costs for children with allergies, driven by medications and healthcare visits.	Food allergies impose a substantial economic burden on families.	Relies on parent-reported data for cost estimates, which may introduce recall and reporting bias. The study focuses on Sweden, limiting its generalizability to other countries. The analysis does not fully account for indirect societal costs.
Suratannon N. et al. [5]	Cost-effectiveness of therapeutic infant formulas for cow’s milk protein allergy management	2023	To evaluate the cost-effectiveness of different therapeutic formulas for managing CMPA in Thailand.	EHCF + LGG is the most cost-effective option for CMPA management.	Analytic decision model	Simulated symptoms and outcomes over 36 months using cost and efficacy data from Thailand.	Infants with CMPA receiving different therapeutic formulas.	EHCF + LGG had the lowest cost and highest effectiveness, saving costs compared to SPF, EHWF, and AAF.	EHCF + LGG is the most cost-effective option for managing CMPA in Thailand.	The cost-effectiveness model is based on expert consensus rather than real-world healthcare data. The study is specific to Thailand, limiting generalizability to other regions. Funded by a commercial entity, which may present potential conflicts of interest.
Sladkevicius E. et al. [24]	Resource implications and budget impact of managing cow milk allergies in the UK	2010	To assess the healthcare resource use and costs of managing CMPA in the UK.	CMPA imposes a substantial healthcare burden in the UK.	Economic modeling study	Used healthcare databases and interviews to model costs for a cohort of infants.	Cohort of 18.350 infants with CMPA in the UK.	Estimated costs were GBP 1381 per patient annually; total annual cost was GBP 25.6 million.	Improved management could reduce the healthcare burden of CMPA.	Uses a budget impact model rather than real-world patient-level data. Focused on the UK NHS, limiting applicability to other healthcare settings. The study does not account for long-term healthcare costs beyond the first year of management.
Sladkevicius E. et al. [38]	Modeling the health economic impact of managing cow milk allergy in South Africa	2010	To evaluate the economic impact of managing CMPA in South Africa.	Managing CMPA imposes significant costs on both healthcare systems and parents.	Economic decision model	Simulated costs over 12 months for public and private sectors based on interviews with clinicians.	Annual cohort of newly diagnosed CMPA infants in South Africa.	Annual cost for insurers: ZAR 22.1 million; cost for parents: ZAR 489.1 million. Costs driven by clinical nutrition and dermatological drugs.	CMPA imposes a significant socio-economic burden in South Africa.	Economic modeling study based on expert interviews rather than real-world clinical data. The study does not account for long-term patient outcomes. Limited applicability beyond South Africa due to healthcare system differences.
Paquete T.A. et al. [39]	Cost-effectiveness of infant hypoallergenic formulas to manage cow’s milk protein allergy in France	2022	To evaluate the cost-effectiveness of hypoallergenic formulas for managing CMPA in non-breastfed children in France.	EHCF + LGG is the most cost-effective strategy compared to AAF, EHWF, and RHF.	Trial-based decision analytic cohort model	Simulated clinical outcomes and costs over 3 years using data from French clinicians and national databases.	Cohort of non-breastfed infants with IgE-mediated CMPA in France.	EHCF + LGG showed the lowest total costs and highest immune tolerance and symptom-free rates compared to other formulas.	EHCF + LGG is the dominant strategy for managing CMPA in France, combining clinical benefits and cost savings.	Economic evaluation based on non-randomized trial data, which may introduce selection bias. The study focuses on France’s healthcare context, limiting broader applicability. Funding from industry sources may introduce a conflict of interest.
Sladkevicius E. et al. [40]	Budget impact of managing cow milk allergy in the Netherlands	2010	To assess the resource implications and budget impact of managing CMA in the Netherlands from the perspective of healthcare insurers.	What are the resource implications and budgetary impact of managing CMA in the Netherlands? Hypothesis: Managing CMA significantly impacts healthcare resources and costs.	A computer-based decision model using TreeAge Pro 2007 to depict treatment pathways for CMA sufferers up to 1 year of age.	Systematic literature review, clinician interviews, and decision modeling incorporating clinical outcomes and resource utilization estimates.	A cohort of 4.382 newly diagnosed CMA sufferers in the Netherlands, based on the estimated annual birth cohort and CMA incidence.	Expected healthcare cost per CMA infant: EUR 2.567. Total expected cost for 4.382 infants: EUR 11.28 million (72% attributed to eHF). Time to symptom resolution: 30 days (on average).	CMA imposes a significant economic burden on the Dutch healthcare system. Using double-blind placebo-controlled challenges could increase costs by ~16%.	Budget impact analysis using modeled assumptions rather than empirical patient data. The study focuses on Dutch healthcare policies, limiting transferability to other settings. Sensitivity analyses may not fully capture real-world cost variability.

CMA: cow’s milk allergy; CMPA: cow’s milk protein allergy; IgE: immunoglobulin E; EHCF + LGG: extensively hydrolyzed casein formula plus Lactobacillus rhamnosus GG; EHWF: extensively hydrolyzed whey formula; eHF: extensively hydrolyzed formula; SPF: soy protein-based formula; AAF: amino acid-based formula.

**Table 2:** Economic burden of CMPA across different healthcare systems.

Country	Year	Average Annual Cost Per Family (USD)	Estimated Total Cost for Healthcare System (Million USD)	Main Cost Drivers
**United Kingdom**	2010	1836.73	34.05	Hypoallergenic formulas, diagnostic tests
**United States**	2022	2625.0	52.5	Specialist consultations, lack of insurance coverage
**Italy**	2015	1998.0	33.3	Hypoallergenic formulas, nutritional support
**France**	2022	2100.0	29.4	Hypoallergenic formulas, hospitalizations
**Australia**	2009	9064.25	9.06	Specialized clinical nutrition preparations
**Brazil**	2016	1332.0	11.1	Limited access to hypoallergenic formulas
**South Africa**	2010	5320.0	29.39	High costs of private treatments
**Indonesia**	2022	945.0	5.25	Limited access to hypoallergenic formulas
**Thailand**	2012	1024.0	6.14	High cost of diagnostic tests
**Netherlands**	2010	3414.11	15.0	High cost of hypoallergenic formulas

All costs have been converted to USD.

## Data Availability

No new data were created or analyzed in this study. Data sharing is not applicable to this article.

## Abbreviations

CMPA	Cow’s Milk Protein Allergy
CMA	Cow’s Milk Allergy
IgE	Immunoglobulin E
eHF	Extensively Hydrolyzed Formula
AAF	Amino Acid-Based Formula
EHCF + LGG	Extensively Hydrolyzed Casein Formula plus *Lactobacillus rhamnosus* GG
EHWF	Extensively Hydrolyzed Whey Formula
SPF	Soy Protein-Based Formula
RHF	Rice Hydrolyzed Formula
LGG	*Lactobacillus rhamnosus* GG
FGIDs	Functional Gastrointestinal Disorders
IBD	Inflammatory Bowel Diseases
EoE	Eosinophilic Esophagitis
DBPCFC	Double-Blind Placebo-Controlled Food Challenge
PPP	Purchasing Power Parity
NHS	National Health Service
PRISMA	Preferred Reporting Items for Systematic Reviews and Meta-Analyses
USD	United States Dollar
DRACMA	Diagnosis and Rationale for Action against Cow’s Milk Allergy
WAO	World Allergy Organization
